# Forty Years of HIV Research in French Guiana: Comprehend to Combat

**DOI:** 10.3390/pathogens13060459

**Published:** 2024-05-28

**Authors:** Mathieu Nacher, Aude Lucarelli, Astrid Van-Melle, Célia Basurko, Sébastien Rabier, Malorie Chroum, Thiago Santana, Karine Verin, Ketty Bienvenu, Myriam El Guedj, Tania Vaz, Hawa Cisse, Loïc Epelboin, Paul Le Turnier, Philippe Abboud, Félix Djossou, Roger Pradinaud, Antoine Adenis, Pierre Couppié

**Affiliations:** 1 Comité de Coordination Régionale de la Lutte contre le VIH et les IST, Centre Hospitalier de Cayenne, 97300 Cayenne, French Guiana; aude.lucarelli@ch-cayenne.fr (A.L.); sebastien.rabier@ch-cayenne.fr (S.R.); malorie.chroum@ch-cayenne.fr (M.C.); thiago.santana@ch-cayenne.fr (T.S.); karine.verin@ch-cayenne.fr (K.V.); ketty.bienvenu@ch-cayenne.fr (K.B.); antoine.adenis@ch-cayenne.fr (A.A.); 2 Centre d’Investigation Clinique INSERM 1424, Centre Hospitalier de Cayenne, 97300 Cayenne, French Guiana; astrid.van-melle@ch-cayenne.fr (A.V.-M.); celia.basurko@ch-cayenne.fr (C.B.); 3 Département Formation Recherche en Santé, Université de Guyane, 97300 Cayenne, French Guiana; pierre.couppie@ch-cayenne.fr; 4 Institut Santé des Populations en Amazonie, Centre Hospitalier de Cayenne, 97300 Cayenne, French Guiana; 5 Hôpital de Jour Adultes, Centre Hospitalier de Cayenne, 97300 Cayenne, French Guiana; 6 Service des Maladies Infectieuses et Tropicales, Centre Hospitalier de Cayenne, 97300 Cayenne, French Guiana; loic.epelboin@ch-cayenne.fr (L.E.); paul.leturnier@ch-cayenne.fr (P.L.T.); philippe.abboud@ch-cayenne.fr (P.A.); felix.djossou@ch-cayenne.fr (F.D.); 7 Service de Dermatologie Vénéréologie, Centre Hospitalier de Cayenne, 97300 Cayenne, French Guiana; roger.pradinaud@orange.fr

**Keywords:** HIV, French Guiana, AIDS, epidemiology, research, public health, historical review

## Abstract

The drivers of the HIV epidemic, the viruses, the opportunistic infections, the attitudes and the resources allocated to the fight against HIV/AIDS, vary substantially across countries. French Guiana, at the crossroads between Amazonian South America and the Caribbean, constitutes a singular context with poor populations and rich country health funding, which has allowed researchers to gather lots of information on the particulars of our epidemic. We aimed to focus on the little known story of forty years of HIV research in French Guiana and emphasize how local research intertwined with public health action has yielded continuous progress, despite the difficult social conditions of the affected population. We searched Web of Science and associated local experts who worked through much of the epidemic in selecting the most meaningful products of local research for clinical and public health outcomes in French Guiana. Research tools and facilities included, from 1991 onwards, the HIV hospital cohort and the HIV-histoplasmosis cohort. Ad hoc studies funded by the ANRS or the European Regional Development fund shed light on vulnerable groups. The cumulative impact of prospective routine collection and focused efforts has yielded a breadth of knowledge, allowing for informed decisions and the adaptation of prevention, testing and care in French Guiana. After this overview, we emphasize that the close integration of research and public health was crucial in adapting interventions to the singular context of French Guiana.

## 1. Introduction

French Guiana is a French territory located in South America, between Brazil and Suriname. Although it has featured a French administration akin to the rest of France since 1946 [1] and a universal health system, it is quite different from mainland France: it is a multicultural territory with ancestries from all continents; its history is marked by different waves of immigration, with populations trying to improve their economic conditions in this morsel of France—a third of the population and nearly half of adults are foreigners [2]; it is marked by widespread poverty with, still today, over half the population living under the French poverty threshold [3]; population growth is the highest in Latin America, driven by a high birth rate and immigration, increasing 5-fold from around 60,000 in the late 1970s to 300,000 today [4]. The exposome in French Guiana and the immunogenetic make-up of the population are also quite different from mainland France [5]. In this context, the epidemiologic transition is still incomplete, with a mix of causes of death before 65 years—notably AIDS deaths—that is more reminiscent of low- and middle-income countries than of mainland France [6].

**Scheme 1:**
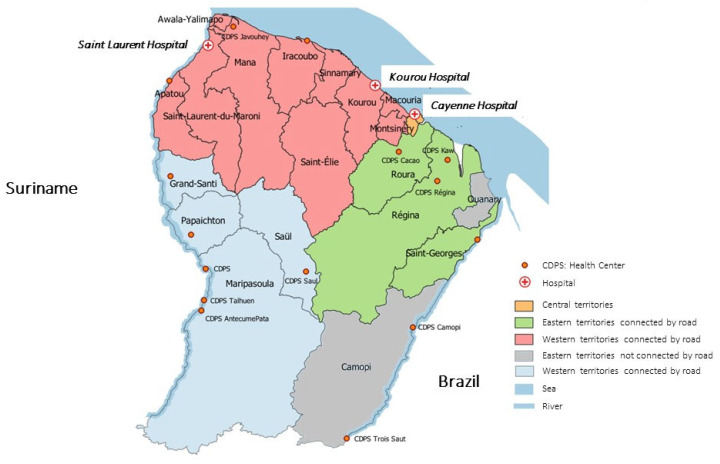
French Guiana and the health structures where initial HIV care can be delivered.

When the AIDS epidemic was revealed in 1981 in the USA [7] and when HIV was described in France in 1983 [8], we now know that, despite the remoteness of the territory, HIV was already in French Guiana. The shock of this pandemic led to unprecedented mobilization of all sectors of society and fueled spectacular scientific progress in understanding and treating HIV, progress that eventually, but not completely, percolated throughout the world. While the global narrative is well known, the particulars in remote parts of the world are not. Indeed, the main drivers of the epidemic, the viruses, the opportunistic infections, the attitudes and the resources allocated to the fight against HIV/AIDS, vary substantially across countries. French Guiana, at a crossroads between Amazonian South America and the Caribbean, constitutes a singular context with poor populations and rich country health funding, which has allowed researchers to gather plenty of information on the particulars of our epidemic. Scheme 1 represents French Guiana, its hospitals and health centers, where most patients living with HIV are referred, in which they benefit from the same treatments and biological explorations as they would in mainland France.

World AIDS day often serves as a reminder that in France, French Guiana is the French territory with the greatest incidence and prevalence of HIV and AIDS, and the epidemic is different, affecting as many women as men in the context of frequent heterosexual multiple sexual partnerships. But this snapshot does not convey the local complexity.

**Figure 1:**
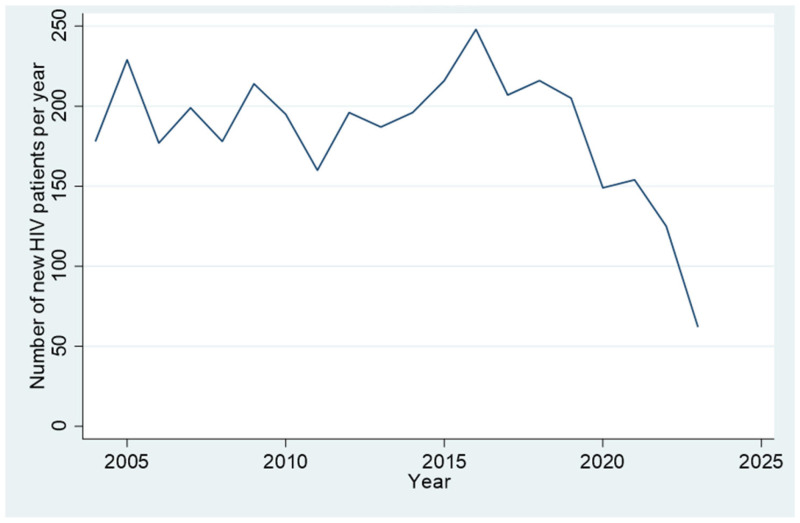
Annual number of new patients with HIV in French Guiana.

At a time when HIV/AIDS research may become detached from the HIV/AIDS coordination in France, the historical overview emphasizes that research and public health action have been and remain synergistic for the benefit of concerned populations. A single line graph with the annual number of new HIV patients could serve as a summary of the epidemic dynamics (Figure 1) in French Guiana, but the details behind this curve are well worth describing.

In the present narrative review, we wish to shed light on the little-known story of HIV in French Guiana and emphasize how local research intertwined with public health action allowed researchers to yield continuous progress, despite the difficult social conditions of the affected population. We first show how the first studies in the 1980s captured the singularity of the epidemic; we then review the vulnerable groups in French Guiana and the epidemic drivers that were targeted by prevention actors; the epidemic in French Guiana is singular, but we show that AIDS in this Amazonian territory encompasses a different mix of opportunistic pathogens, knowledge that is crucial to care for those with advanced HIV infection in French Guiana and beyond; finally, we show how modern phylogenetic approaches confirm past studies and shed new light on the history of the HIV epidemic in French Guiana, and we synthesize the progress made and its indebtedness to local research, guiding public health action. 

## 2. Materials and Methods

**Figure 2:**
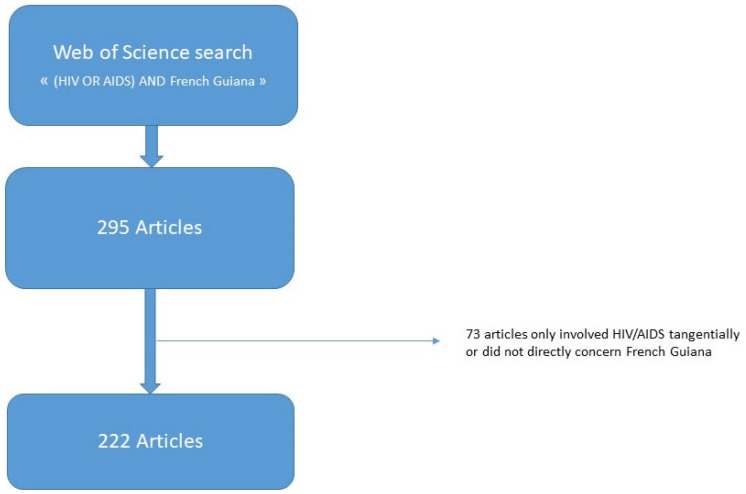
Flowchart of the Web of Science search.

We searched Web of Science with the French Guiana AND (HIV OR AIDS) keywords and associated French Guianese local experts who worked through much of the epidemic in selecting the most meaningful products of local research for clinical and public health outcomes in French Guiana. Figure 2 shows a flowchart of the search. 

**Figure 3:**
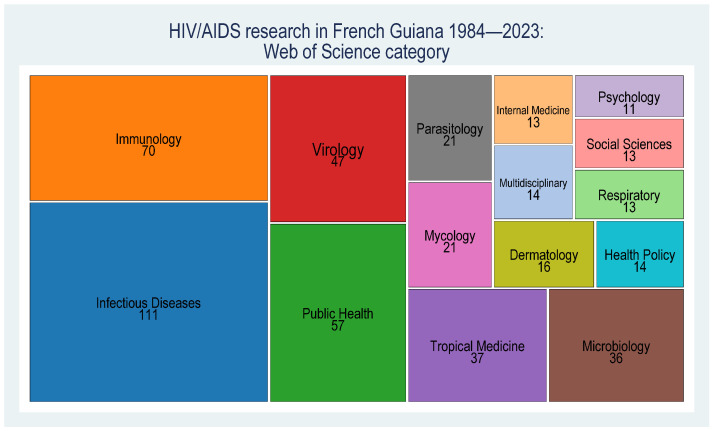
Web of Science classification of research on HIV or AIDS in French Guiana.

**Figure 4:**
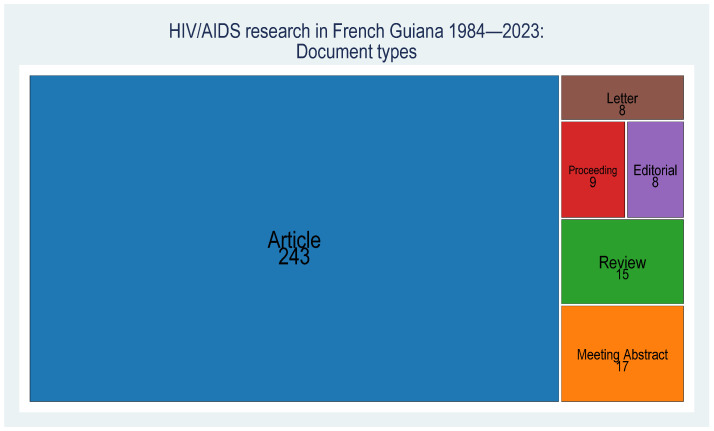
Web of Science type of research documents on HIV/AIDS in French Guiana.

Among the 295 articles returned by the query, 73 articles only briefly mentioned HIV or AIDS in French Guiana; some were mostly focused on HTLV-1 or other diseases, while others were on HIV but without any reference to French Guiana. The research themes and types of research documents are shown in Figure 3 and Figure 4. Supplementary Figures S1 and S2 show the VOSviewer analysis (version 1.6.20 available at https://www.vosviewer.com/).

Because the territory has developed rapidly in the past 40 years in terms of research capacity, we emphasized important local and international milestones in Supplementary Figure S3. We organized the results chronologically and thematically.

Research tools and facilities included, from 1991 onwards, the HIV hospital cohort, which is collected by trained research technicians and is part of the French Hospital Database on HIV. In 1999, the HIV-histoplasmosis cohort was initiated by Professor Pierre Couppié and is still ongoing. Since 2003, the HIV mandatory declaration reports have also allowed us to track the epidemic trends. Since 2018, the Système National des Données de Santé (SNDS) has compiled data from health insurance, hospital information systems and death certificates, which provides a glimpse of HIV care beyond hospital cohorts. Ad hoc studies funded by the Agence Nationale de Recherche sur le SIDA et les Hépatites (ANRS) or the European Regional Development fund shed light on vulnerable groups. Finally, numerous MD or PhD thesis candidates focused on a wide range of topics related to HIV/AIDS. The cumulative impact of prospective routine collection and focused efforts has yielded a breadth of knowledge, allowing for informed decisions and the adaptation of prevention, testing and care in French Guiana.

## 3. Results

### 3.1. The 1980s: HIV/AIDS Is There and It Is Not the Same as in Mainland France

Given Pasteur Institute’s sustained focus on HTLV-1 in French Guiana, bio-banked samples from regular serologic surveys allowed us to test for HIV early on (then called HTLV-III). Hence, in 1984, it was shown that 15/211 samples from Haitian immigrants were positive for a semi-purified HTLV-III ELISA (7.1%, 7 from males, 8 from females) [9]. However, Western blot analysis only confirmed 6/211 positives (2.8%, 95%CI = 1–6%). The six individuals from whom these samples were obtained had emigrated to French Guiana 2–9 years previously [9]. By the end of 1987, over 100 cases of AIDS had been reported in a population of circa 100 000 inhabitants at the time, and transmission was mostly heterosexual.

The next published studies reported on HIV/AIDS cases among pregnant women and the high vertical transmission rate, with only 40% percent of infants testing negative [10]. The study also showed that most (40/44) of these pregnant women were Haitian nationals; the first retrospectively identified AIDS case was observed in 1979 in a Haitian pregnant woman.

Since AIDS was often associated with dermatological manifestations, and since it is sexually transmitted, HIV/AIDS care was centered around the department of dermatology-venereology, which begun to systematically test all herpes zoster or prurigo cases for HIV, revealing that over a third of such cases were HIV-positive [11,12,13]. This allowed us to see that, over time, the proportion of positive HIV serologies among persons consulting for herpes zoster or prurigo remained stable among Haitian and French Guianese patients, while there was a surge in other nationalities during the 1990s, reflecting the epidemic spread to persons from Suriname, Guyana and the Dominican Republic.

Because the interior regions of French Guiana are not accessible by road, transmission was delayed there for up to a decade. The HIV epidemic, thus, started to spread along the coastal cities, but, eventually, the HIV prevalence among pregnant women caught up with that of women in the main coastal cities, presumably driven by boatmen, a bridging group of highly mobile men with multiple sex partners [12,14]. However, the most remote areas in the interior, areas where Amerindian populations live, still require special prefectorial authorizations, and, thus, sexual networks remain isolated [15], and the spread of HIV has failed to entrench itself there.

Overall, the main drivers fueling the epidemic in French Guiana were quite different from mainland France and consisted of multiple concomitant heterosexual partnerships and transactional sex in a context of widespread poverty. The social and behavioral consequences of crack cocaine—selling sex in order to use and sometimes buying sex after using—led to substantial transmission in this group, with one study finding 14% of HIV-positive users at an addiction clinic in Cayenne [16]. These were the initial insights gained from early works in French Guiana.

### 3.2. Running after the Epidemic and Improving the Cascade of Care

The epidemic growth in the 1990s translated into circa 200 additional HIV diagnoses each year, with over 30% being diagnosed with a CD4 count under 200 per mm^3^, a figure that seemed to remain depressingly stable, whatever was attempted to increase early diagnosis [17]. In the Western border town of Saint Laurent du Maroni, year after year, 50% were tested below 200 CD4, a situation that reflected the fact that across the river, in Suriname, patients were only treated when CD4 counts dropped below 200 per mm^3^ until 2018 [18].

Efforts to understand late diagnosis showed that foreigners, males and older age groups were most likely to be tested at the stage of advanced HIV [19]. In addition, the rapid growth of hospital cohorts and the rate of follow-up interruptions remained high, notably in the first 6 months after diagnosis, with half of patients having interrupted follow-up by year 5 [20]. Overall, the permanent follow-up interruption rate was 17.2 per 100 person-years. Younger age groups, foreigners, persons from Western French Guiana, those with initial CD4 counts under 500/mm^3^ and untreated patients were significantly more likely to be permanently lost to follow-up [20].

In this context of social vulnerability and frequent stigma, insufficient adherence levels led to suboptimal virological suppression and cascade of care [21]. However, with time, intensification and diversification of HIV testing, reinforced therapeutic education and even sometimes directly observed antiretroviral therapy by nurses at the patient’s home led to an 85 × 91 × 91 cascade of care by 2017 [22].

The SNDS data showed that, with the simplicity of new treatments, there has been a secular trend of patients, everywhere and of all social origins, to be exclusively followed by their general practitioners [23]. As we shall see in the modelling section, testing efforts, simple new antiretroviral regimens, adherence support, reduced transmission and the ever-increasing portion of virologically controlled persons living with HIV led to a decline in the reservoir of undiagnosed infections.

### 3.3. Understanding Our Vulnerable Groups to Adapt Prevention

#### 3.3.1. The General Population

The core epidemiologic features of our epidemic reflect different drivers than in mainland France, and it was, thus, essential to describe the knowledge, attitudes and practices of the different segments of the population, notably of the most at-risk populations. Hence, the regional health observatory of Ile de France conducted two knowledge attitudes, behaviors and practices studies in the general population in 2004 and 2011 that allowed for comparisons with similar studies in mainland France [24,25]. This revealed a number of stable salient features in French Guiana: sexual relations were often more precocious, where the proportion of men resorting to paid sex was about double of that of mainland France; multiple concurrent partnerships were far more frequent in French Guiana; knowledge of transmission modes and means to protect oneself were much lower in French Guiana, notably among the poorest; finally, stigmatizing attitudes were more frequent in French Guiana than in mainland France. In this sexual network context, women who could not negotiate prevention were at risk of acquiring HIV from their partner. Moreover, in the context of stigma and social precariousness, revealing one’s HIV-positive status has always been difficult, leading to substantial transmission from persons aware of their diagnosis to others who were not [26]. Hence, among pregnant women, in the 2000s, three-quarters had not told the father of their present child that they were infected; we also showed that the growth in the number of new patients was correlated with the number of current patients who did not achieve control of virological replication, suggesting that, despite the availability of free condoms, protection was far from systematic, with consequences [27]. In addition, studies were launched based on some of the most at-risk populations, sex workers, teenagers, men who had sex with men (MSM), crack cocaine users, immigrants, boatmen, prisoners and persons living on the Maroni river, where prevalence went from zero to over 1% in a decade.

#### 3.3.2. Sex Workers

The survey showed that 97% of female sex workers declared systematically using condoms with their clients, a very high rate, but only 45% did so with their regular partner [28]. However, this study did not cover occasional transactional sex or transactional sex in addicts, where protection levels were probably far worse.

#### 3.3.3. Teenagers

Surveys in schools showed that early sexual intercourse was common and that 16% reported an unwanted or forced first intercourse [29]. Knowledge on HIV transmission and prevention was poor but increased with age; stigmatizing attitudes were widespread but decreased with age and improved knowledge.

#### 3.3.4. Immigrants

In a study of 1039 immigrants, 39% had engaged in risky sex. Among males, half had multiple sexual partnerships (versus 25% in the general population), 35% had concurrent relationships (versus 14% in the general population), 15% had transactional sex relations (versus 4.9% in the general population) and 7.1% did not use a condom during their last sexual encounter (versus 4.5% in the general population) [30]. Over half of the migrant population declared having sex with persons of a different nationality, suggesting the bridging of different sexual networks.

#### 3.3.5. Illegal Gold Miners

The soil of the interior of French Guiana is rich in gold and attracts numerous illegal gold miners who live in the deep forest and establish settlements, where sex work is common. In this context, some studies among gold miners have shown that the prevalence of HIV could reach levels greater than 1% [31,32], and that they were often diagnosed very late [33].

#### 3.3.6. Men Who Have Sex with Men (MSM)

Although the epidemic was essentially driven by heterosexual sex, with an even male/female ratio, high-risk behaviors were highly prevalent. Hence, 233 respondents, 73%, reported multiple sex partnerships in the past year, and 22% had been subjected to forced intercourse during their lifetime. It was notable that over 20% of persons engaged in transactional sex, and 23% reported not using a condom with a commercial partner at their last sexual encounter [34]. For most recent oral sex, 64% had not used a condom, and for anal sex, it was 31%. Alcohol was often consumed (47%) before transactional sex. By contrast, HIV testing was common (94%) and mostly in the past year (80%) [34].

#### 3.3.7. Populations Living on the Maroni River

Given the rapid spread of the epidemic along the Maroni river in the 1990s, a specific study was conducted to better understand why. The survey of 896 individuals showed that first sex often occurred before 15 years (a third of Maroon women and half of Amerindian women); that it was often forced (6.6% of Maroon women vs. 11.4% of Amerindian women); concurrent multiple sex partnerships were more frequent, mostly in Maroons, than in the general population; women, but not men, were less likely to report using condoms on the Maroni; Amerindians were less likely to use condoms [35]. Only a quarter had used a condom at their last sexual intercourse. Stigma and discrimination were frequent [36], but over 90% of people were very willing to take an HIV test. Of uncertain significance, we must report that a substantial proportion of men had inserted penile implants (16.6% of maroons and 9.3% of Amerindians), which may interfere with condom use [35].

#### 3.3.8. Boatmen

Among Maroni river boatmen, nearly a quarter had never done an HIV test, despite a long list of sexual risks: first sexual activity was precocious and unprotected; over eighty percent declared multiple sexual partners and half declared having paid for sex in the past year; 30% declared having had a sexually transmitted infection in the past year. When asked about their last intercourse, only 57% had used a condom [14]. All the above, in conjunction with the high proportion of late HIV diagnoses—half diagnosed with advanced HIV—explain why this highly mobile population was suspected to be an important bridging group in the inland spread of the epidemic.

#### 3.3.9. Crack Cocaine Users

Among crack users, sexual risks were high: about a third of patients never used condoms for casual sex, including during paid sex [28]. Further, 57% of those living in a couple had multiple sexual partnerships, and nearly a quarter reported having forced sex. HIV knowledge was poor, and HIV testing was insufficient. Unsurprisingly, prevalence in the addiction clinic in Cayenne [16] was 14.3%, and the incidence of AIDS-defining events was almost 4-times that of those who did not use crack cocaine [37]. Finally, women who used crack were identified as particularly at risk of dying [38].

#### 3.3.10. Prisoners

HIV prevalence at the penitentiary center of French Guiana has oscillated around 4% for decades. Social precariousness and a high prevalence of mental health problems are known risk factors for HIV that are overwhelmingly prevalent at French Guiana’s sole penitentiary center. In French Guiana, inmates also frequently insert penile implants in their foreskin, which makes condom use challenging and may cause tissue damage during intercourse [39]. The most striking feature is, however, the very high mortality (4.2 per 100 person-years) of persons living with HIV after prison release, emphasizing that while their health is usually improved while incarcerated, the return to their initial social conditions degrades their treatment and care [40].

#### 3.3.11. Information for Action

All the above information was integrated in large communication campaigns using multiple channels (video clips and songs, TV, radio, posters, road signs, T shirts…), funded and organized by the national institute for prevention and health education. Furthermore, and perhaps more importantly, local NGOs used this strategic information to optimize their field preventive actions. Finally, and recently, pre-exposure prophylaxis was scaled up to cover the most at-risk populations.

#### 3.3.12. A Noticeable Data Collection Gap

While researcher-driven efforts were made to describe the knowledge and attitudes of different vulnerable population groups, specific seroprevalence surveys were generally never conducted (apart from crack cocaine users or inmates), in contrast to what was being recommended by UNAIDS’s “know your epidemic know your response” emphasis on the biological surveillance of vulnerable groups [41]. NGOs attending to these vulnerable groups generally did not produce statistics on the seroprevalence of HIV among men who have sex with men, or sex workers, or immigrants. Health authorities rarely funded or requested such data. The most likely explanation for this gap regarding an important indicator was the reluctance to produce potentially stigmatizing statistics. This makes it difficult to track, in great detail, how the epidemic evolved in different subgroups.

### 3.4. Revealing the Face of “Amazonian AIDS”

In French Guiana, the presence of cutaneous leishmaniasis led dermatologists to conduct smears of cutaneous or mucous lesions, to stain them and to read the slides as part of a normal consultation [42]. In this context, when the first AIDS cases arrived with umbilicated facial pustules, or with oral lesions, dermatologists scraped and stained them, finding the invasive fungal pathogen *Histoplasma capsulatum*, an AIDS-defining infection since 1987. So, from the very onset of the epidemic, clinicians were aware of the presence of histoplasmosis [43], but these cutaneo-mucous cases were generally very disseminated infections that were ultimately spreading to the skin [44]. Case fatality was 40%.

The implementation of fungal culture in the late 1990s revealed that incidence was actually much greater than initially observed and, with time, the number of diagnoses surged and cutaneo-mucous forms became exceptional, while case fatality gradually declined [45]. This led to the constitution of the histoplasmosis cohort, which is still ongoing and has been precious in the detailed study of this disease.

The HIV hospital cohort, which compiles clinical and therapeutic data of all HIV-diagnosed patients, showed that in French Guiana, the most frequent AIDS-defining infection was, by far, disseminated histoplasmosis, and this was still the case in 2020 [44,45]. It was also the first cause of death. Hence, in Saint Laurent du Maroni, one of the three biggest cities of French Guiana, and located on the Maroni river bordering Suriname, 42% of HIV patients with less than 200 CD4 per mm^3^ were hospitalized with fever, 85% of those with less than 50 CD4 per mm^3^ and had disseminated histoplasmosis: “First think histoplasmosis!” [46]. Another study compiling cases of hemophagocytosis among persons with HIV was essentially due to disseminated histoplasmosis—there, again, first thought to be histoplasmosis [47].

The HIV cohort allowed us to quantify the overall incidence of dissemination at 1.5 per 100 person-years [48]; however, when stratifying on CD4 counts, the incidence rate exceeded 10 per 100 person-years for persons with less than 50 CD4 per mm^3^ [49], and there were seasonal fluctuations in incidence, peaking during the dry season, which emphasized that cases were probably often new infections [50]. The HIV cohort also revealed that disseminated histoplasmosis was also associated with immune reconstitution disease [51,52]. Finally, the HIV hospital cohort showed that antiretroviral treatment itself was independently associated with a 5-fold decrease in case fatality [49].

All these insights from an Amazonian territory were important to quantify the burden of histoplasmosis among the HIV cohort. Saying that histoplasmosis is the number one opportunistic infection is a way of framing the problem—the view from the cohort—that has far more implications in terms of burden of disease than saying that most cases of disseminated histoplasmosis also have HIV (the view from the lab). Apart from histoplasmosis, other invasive fungal infections are common, with cryptococcal meningitis as the second fungal pathogen. Another striking difference is that while *Pneumocystis jirovecii* pneumonia has long been the main opportunistic infection in mainland France and other temperate countries, in French Guiana, it only ranked fourth in terms of incidence [45].

Disseminated histoplasmosis often appears in very immunosuppressed persons who may have other opportunistic infections. Since Samuel Darling’s discovery of histoplasmosis over a hundred years ago “…in a case that appeared to be miliary tuberculosis …” it is often said that histoplasmosis presents like a tuberculosis-like illness. Framing the question in this way, in a context where histoplasmosis is more frequent and more lethal than tuberculosis, led to the argument that it is tuberculosis, which is a histoplasmosis illness and if, in this context, a physician was to choose to start treating one disease, it should be histoplasmosis [53]. Furthermore, when comparing confirmed cases of disseminated histoplasmosis with confirmed cases of tuberculosis, the idea that they are similar became questionable, as they are actually often fairly different [54,55].

Disseminated histoplasmosis is proteiform [56], and it may be severe, moderately severe or mild, with fuzzy boundaries between categories. The histoplasmosis cohort allowed us to determine prognostic factors for early death [57], with a recent update and prognostic score [58] to help determine what induction antifungal treatment should be given, itraconazole or liposomal amphotericin b; the cohort also allowed us to describe the various presentations [59,60,61,62,63,64], the proportion of severe forms and its relation with common clinical presentations [44,59,65,66].

There have been debates about the differences in different *Histoplasma* clades in terms of virulence, with an apparently greater virulence of South American isolates. Clinically, this is difficult to demonstrate, given the confounding linked to the level of immunosuppression. Until recently, we were inclined to attribute the proteiform presentations to a combination of acquired immune suppression, immunogenetic make-up and randomness, hence dismissing the possibility that such phenotypic differences reflected genetic differences [67]. However, recent work emphasized the huge genetic variation in *Histoplasma capsulatum*, with four different phylogenetic species isolated within French Guiana, three of which had never been described before [68]. The fact that principal component analyses on the histoplasma cohort data identified four main components [60] begs the question whether the four phylogenetic species have anything to do with this.

### 3.5. Advocating for a Neglected Disease and the First WHO Histoplasmosis Guidelines

Amongst the many details described above, the most important fact is that in this South American territory with an HIV cohort, the number one AIDS-defining infection and the number one cause of death of persons living with HIV is histoplasmosis. This is important because it probably reflects what is happening in other parts of Latin America, and emphasized that, in Latin America, and perhaps far beyond, it is a massively neglected killer of persons living with HIV [69,70,71,72]. The first crude estimates were refined by a detailed burden of disease in a Latin American study, providing, for the first time, a country-by-country estimate of prevalence, incidence and fatalities for both histoplasmosis and tuberculosis [73].

From bottom-up research and advocacy from clinicians, biologists and epidemiologists, the realization of the magnitude of the problem led to advocacy and the mobilization of international health authorities, and now, hopefully, top-down efforts to provide affordable rapid diagnostic tests and effective treatments to all endemic countries. The increasing flow of data from the region, with lobbying from GAFFI (Global Action For Fungal Infections), led to the inclusion of itraconazole in the WHO essential drugs list and then to include histoplasma antigen diagnostic tests in the WHO’s essential diagnostic tests [74].

This is very important because in Latin America, thousands of persons die each year, and it is becoming increasingly apparent that Africa and Asia may also have a substantial burden of histoplasmosis among persons living with HIV [75]. This may seem far from the epidemic in French Guiana, but it is research that has transformed the dermatologists’ clinical acumen into cohorts and indicators that allow us to see what UNAIDS and PAHO, and most Latin American health ministries, failed to see. And it was only 40 years after the first AIDS cases that the arguments provided by research swayed international authorities.

### 3.6. Refining Our Understanding of the Dynamics of the Epidemic: Modeling and Phylogeny

The basic epidemiologic features observed in French Guiana pointed to an epidemic starting in the 1970s, fueled by heterosexual transmission in a context of frequent multiple sexual partnerships and transactional sex in poor immigrant communities. This led to frequent stigmatizing shortcuts, alleging that HIV was a problem of foreigners, not French Guianese.

We used two independent methods to determine whether HIV was indeed mostly imported from other countries: first, a method based on the erosion of CD4 counts [76], and then molecular epidemiology using polymerase sequences to estimate the proportion of infections acquired abroad [77]. Both methods yielded the same conclusion: most infections among foreign nationals were acquired after their arrival in French Guiana, which counters popular beliefs. Furthermore, the CD4 erosion technique suggested that infection was generally acquired in the first years after arrival—half were estimated to have been infected withing 4 years after their arrival in French Guiana [78]—emphasizing the need for active prevention and testing efforts among vulnerable immigrants.

HIV RNA mutates frequently; the comparison of polymerase sequences, thus, allows us to retrace the history of the epidemic. The first conclusion of phylogenetic studies is that the epidemic is akin to what is observed in the Caribbean. Hence, two-thirds of the viruses are of Caribbean type B (B_CAR_), which historically originate from the island of Hispaniola, shared by Haiti and the Dominican Republic [79]. The remaining third mostly consists of B_pandemic_ viruses. However, phylogenetic analysis also emphasizes that these viruses exhibit a “Guiana Shield” profile, which emphasizes the importance of local transmission, where lineages have been repeatedly exchanged, in both directions, between French Guiana and Suriname, reflecting the pendular migration between countries. From the earliest phase of the epidemic, a substantial proportion of the hospital cohort were Haitians. Phylogenetic analyses showed that while immigrants from the Guiana Shield were often likely to cluster within large or medium transmission clusters, Haitian patients were, by contrast, mostly (74%) infected by unclustered lineages (51%) or dyads (23%), which suggests they had acquired HIV before arriving in French Guiana.

The phylodynamic analyses confirmed the major milestones drawn from the simple epidemiologic analysis. The epidemic indeed began in the late 1970s, slowly increasing during the 1980s and accelerating in the 1990s, followed by a plateau [22]. Phylodynamic analyses estimated that the mean number of secondary transmissions (R_0_) ranged between 3.4 and 4.7, which is similar to some estimates in the Caribbean or Central America. However, this is lower than R_0_ estimates in epidemics driven by men who have sex with men in the United States, Cuba, Europe and Hong Kong.

### 3.7. Measurable Success

**Figure 5:**
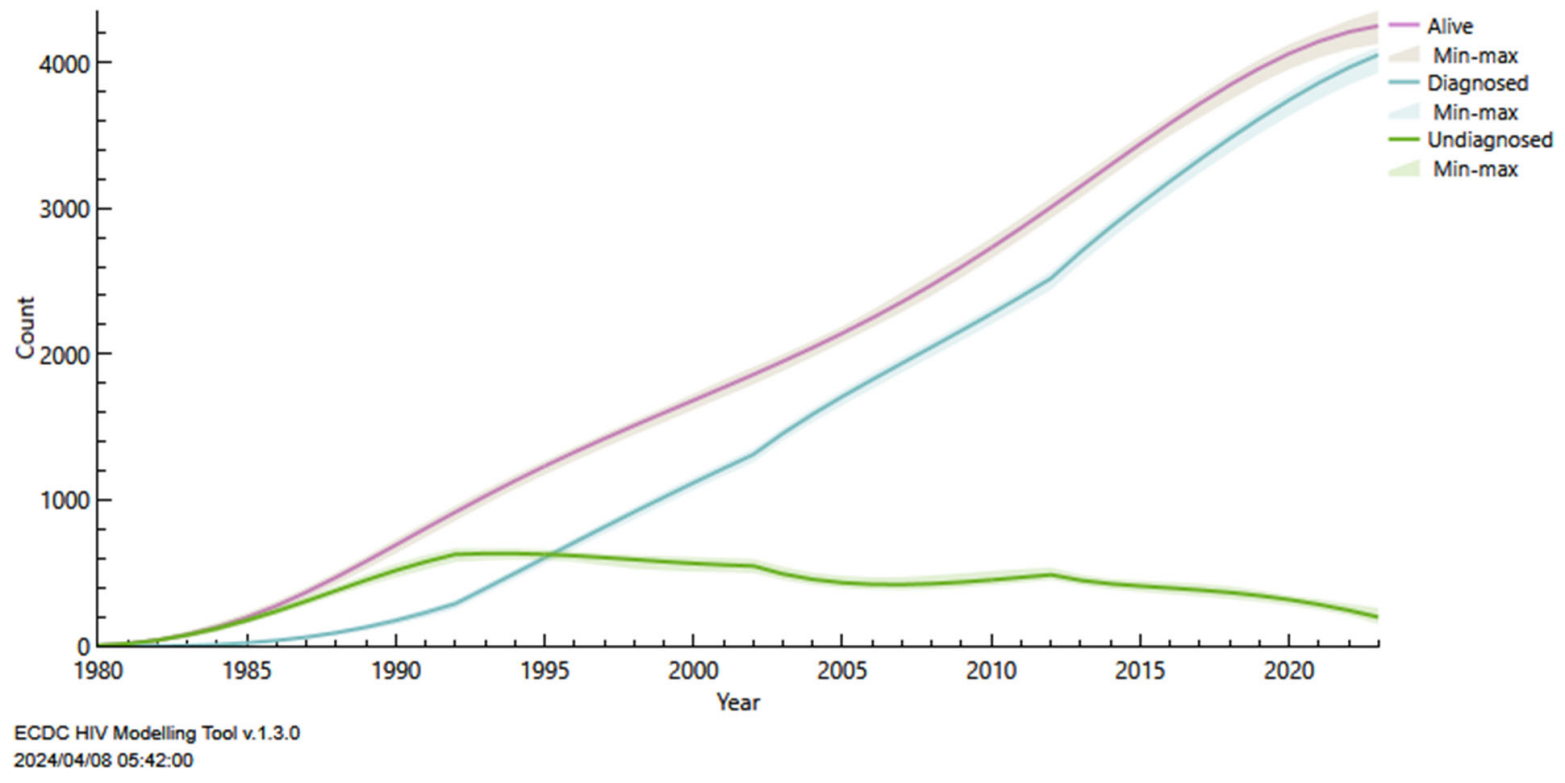
ECDC modelling tool estimates of diagnosed and undiagnosed persons living with HIV in French Guiana.

**Figure 6:**
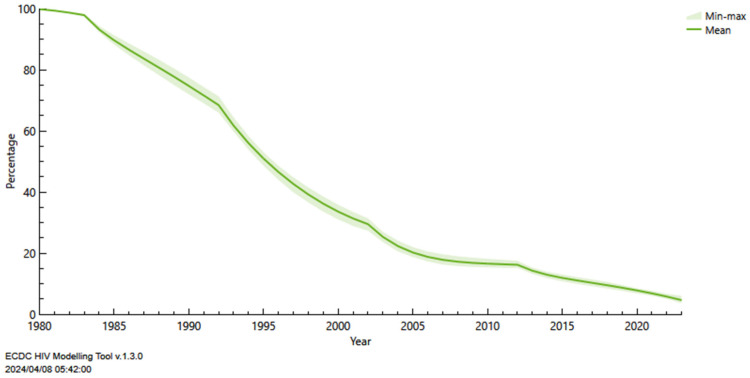
ECDC modelling tool estimates of the evolution of the proportion of undiagnosed persons living with HIV in French Guiana.

Finally, after all this research, after witnessing the decline in new HIV diagnoses (Figure 1), and, at last [17], the decline in the proportion of persons diagnosed with advanced HIV, we modelled the dynamics of the epidemic using the European Centers for Disease Control (ECDC) modelling tool and the incidence method (Figure 5 and Figure 6). This showed that the number of persons living with HIV was greater than 4000, with circa 200 undiagnosed persons, reflecting the continuous meltdown of the number and proportion of undiagnosed infections, around 5%, which means the first target of the 95 × 95 × 95 seems to have been achieved with a 95% × 92% × 94% HIV cascade at the end of 2023 [80].

The fight against HIV entails the efforts of many actors on prevention, testing and care, and all of these have been instrumental. However, although it is correlational, it does seem that putting nearly all HIV-diagnosed persons on effective antiretroviral therapy in French Guiana and neighboring countries was synchronous to the decline of the number of new HIV diagnoses. It does not seem that many of the main epidemic drivers are still present and hard to impinge upon (poverty is worsening, apart from COVID-19’s 2020–2021 lockdowns, sexual and health-seeking behaviors have not changed markedly) so the drastic reduction in the community viral load indeed seems to be the most likely explanation for recent successes. However, the continuous dissemination of information on specific aspects of the epidemic within the public health networks allowed for an agile response to new information. For example, the gradual realization of the importance of histoplasmosis led to a rapid increase in diagnoses and a rapid decrease in case fatality; the realization of the high incidence of follow-up interruption led to a strengthening of the bond between NGOs and outpatient care providers, emphasizing the importance of health mediation.

### 3.8. Reinjecting Knowledge from Research to Optimize Public Health Action

In French Guiana and elsewhere, administrators and NGOs do not usually read, or have access to, scientific articles in English. The echoes of the main international breakthroughs may still percolate to them, but the regional research usually does not. In a small territory, where the actors involved in the fight against HIV are limited in number, the coordinating organizations managing the HIV cohort were generally sufficient to permit the fluid brokering of Public Health knowledge. Since 1985, the Centre d’Information et de Soins de l’Immunodeficience Humaine (center for information and care of human immunodeficiency, CISIH), and since 2008, the Coordination Régionale de lutte contre le VIH (Regional HIV Coordination, COREVIH) have been structures where health professionals, NGOs and public health administrators could exchange the data provided by the region’s HIV cohort. The structures also organized work sessions to tailor interventions based on detailed data on the most at-risk populations. Furthermore, newsletters gave regular feedback about important results.

### 3.9. Limitations

The present study spans 40 years of research to provide a contemporary history of the HIV epidemic in French Guiana. The selection of the chapters of this history may be criticized as they are somewhat arbitrary. However, the selection was performed by persons who have witnessed firsthand the unfolding of the epidemic in French Guiana. Altogether, although the research provides a pointillist view of the epidemiology, some gaps remain in the intricate details of the history. Detailed repeated seroprevalence studies in the most at-risk populations were never performed, and we assume that the spread of the virus went from compartment to compartment, but this was not specifically documented. Finally, although knowledge attitudes and belief studies were conducted, qualitative studies are lacking to shed light on patient narratives about many aspects of HIV and health, an important point to cover if changing behaviors is the aim.

## 4. Conclusions

Naturally the extraordinary progress in antiretroviral treatments, genotyping to choose effective treatments, rapid diagnostic tests, the provision of health insurance and residence permits for the most socially vulnerable have been absolutely essential in reaching 95 × 92 × 94. However, in our singular territory with a singular epidemic, we believe local or regional research has not been trivial. Indeed, it has also been essential to fight the epidemic, to attain undiagnosed patients, to target the most at-risk populations in our context, to frame messages and to adapt our diagnostic and treatment strategies to our pathogen ecosystem when patients fall ill. This research was mostly our responsibility; it cannot be the duty of researchers 7000 km away. To emphasize the dynamism of local research, it is worth noting that there were nearly 30-times more publications per person living with HIV than in mainland France, that is in proportion to the number of HIV-infected persons in each territory. The virtuous circle between public health and research is essential, and we argue that separating the two would lead to an impoverishment of knowledge and public health responses. Indeed, standard activity reports sitting on bookshelves with too much information to grasp would not have noticed histoplasmosis (as UNAIDS and PAHO did) and would not have studied the phylogenetics or dynamics of the epidemic, whereas research always plunged into further detail. The separation between research and public health, while tempting for the administration, may herald an era of data retention or bureaucratic requirements to share data, which would dry up efforts to analyze the details, in general, and specifically for a small singular territory.

## Data Availability

For the graphs, data may be shared upon reasonable request after approval of the Commission Nationale Informatique et Libertés.

## Supplementary Materials

The following supporting information can be downloaded at: https://www.mdpi.com/article/10.3390/pathogens13060459/s1, Supplementary Figure S1. VOSviewer analysis of the publications on HIV/AIDS and French Guiana: by key word. Different colors represent different clusters; Supplementary Figure S2. VOSviewer analysis of the publications on HIV/AIDS and French Guiana: by author. Different colors represent different clusters; Supplemental Figure S3. Milestones of HIV/AIDS research in French Guiana.
