# Supplementary material for: Forty Years of HIV Research in French Guiana: Comprehend to Combat
Source: Pathogens. 2024 May 28;13(6):459. doi: 10.3390/pathogens13060459 (PMC11206598; doi:10.3390/pathogens13060459)
Supplement: Supplementary file 1 [file pathogens-13-00459-s001.zip › pathogens-2991447-supplementary.pdf]

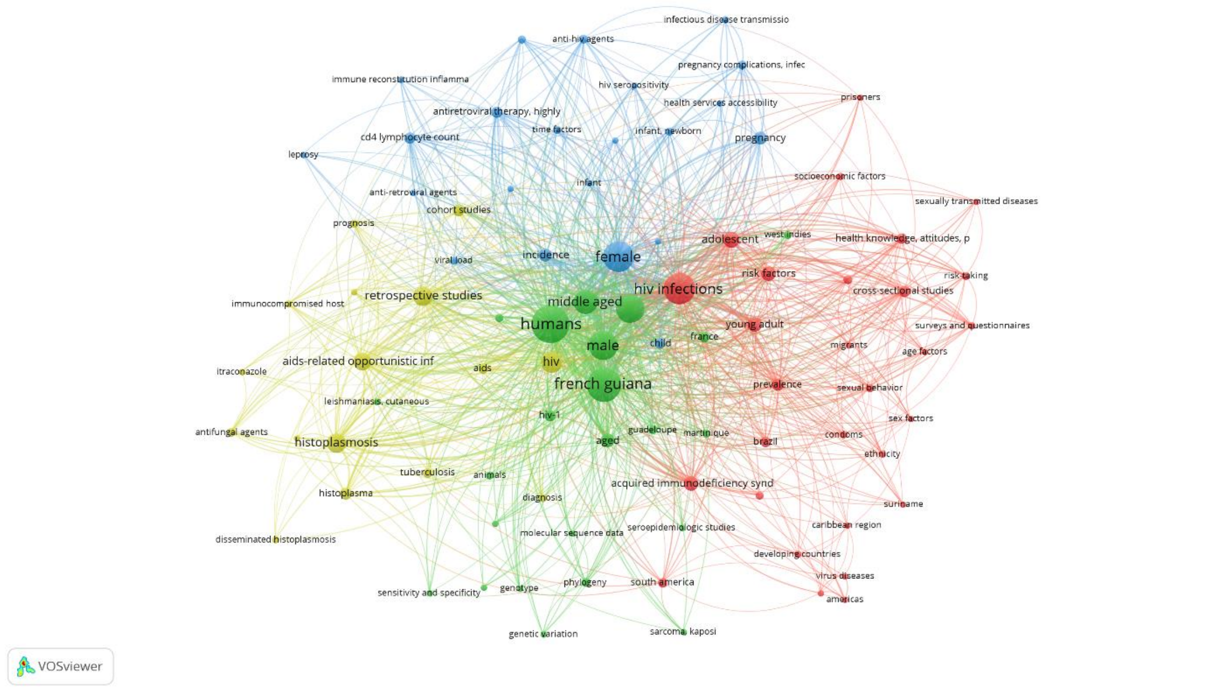

Supplementary Figure S1. VOSviewer analysis of the publications on HIV/AIDS and French Guiana: by key word. Different colors represent different clusters.

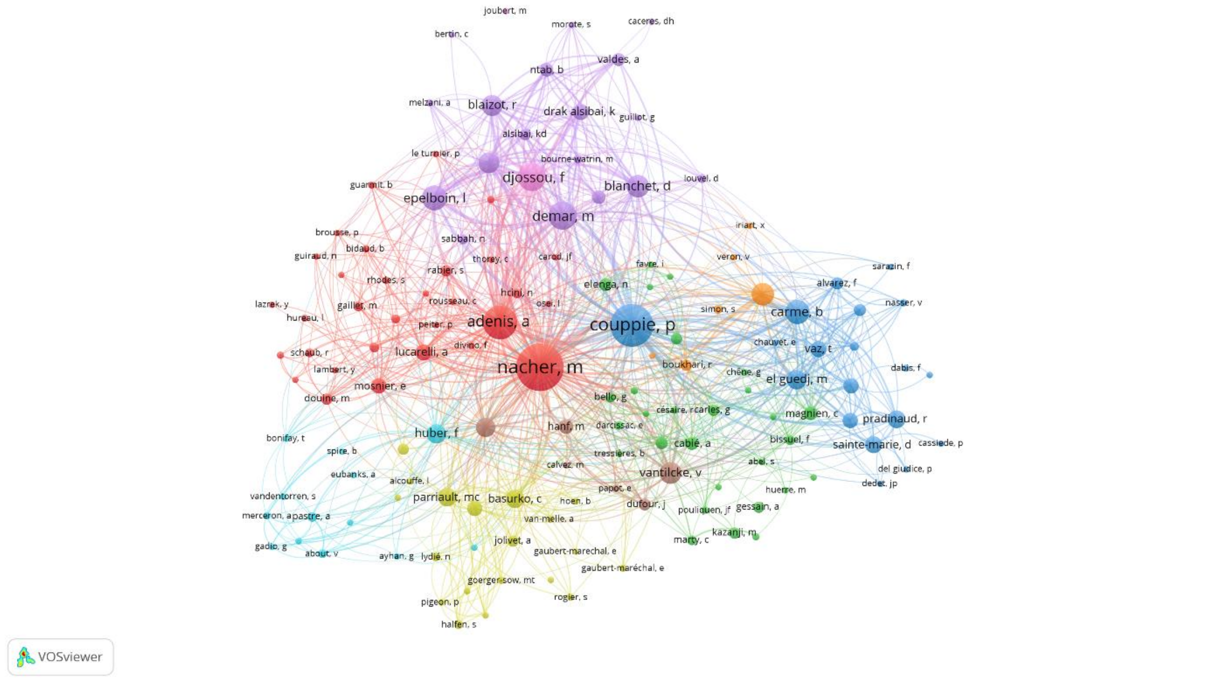

Supplementary Figure S2. VOSviewer analysis of the publications on HIV/AIDS and French Guiana: by author. Different colors represent different clusters.

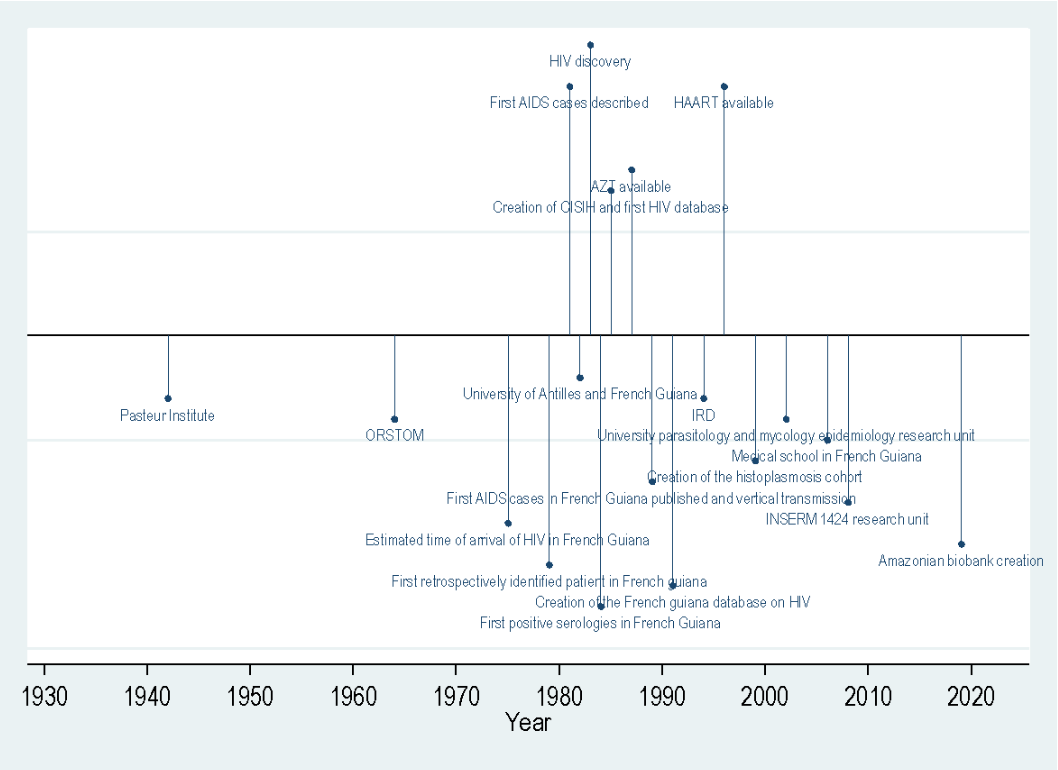

Supplemental Figure S3. Milestones of HIV/AIDS research in French Guiana.

ORSTOM: Office of Scientific and Technical Research Overseas; INSERM: Institute for Scientific and Medical Research; IRD: Institute for Research for Development. CISIH: Center for Information and Care of Immunesuppression (replaced in 2008 by COREVIH (Regional Coordination for the fight against HIV);
